# Porewater salinity reveals past lake-level changes in Lake Van, the Earth’s largest soda lake

**DOI:** 10.1038/s41598-017-00371-w

**Published:** 2017-03-22

**Authors:** Yama Tomonaga, Matthias S. Brennwald, David M. Livingstone, Olga Kwiecien, Marie-Ève Randlett, Mona Stockhecke, Katie Unwin, Flavio S. Anselmetti, Jürg Beer, Gerald H. Haug, Carsten J. Schubert, Mike Sturm, Rolf Kipfer

**Affiliations:** 1 Eawag, Swiss Federal Institute of Aquatic Science and Technology, Department of Water Resources and DrinkingWater, Überlandstrasse 133, 8600 Dübendorf, Switzerland; 2 Atmosphere and Ocean Research Institute, The University of Tokyo, 5-1-5 Kashiwanoha, Kashiwa-shi, Chiba, 277-8564 Japan; 3 Institute of Geological Sciences, University of Bern, Baltzerstrasse 1+3, 3012 Bern, Switzerland; 4 Geological Institute, Swiss Federal Institute of Technology (ETH), 8092 Zürich, Switzerland; 5 Ruhr-University Bochum, Universitätstrasse 150, 44801 Bochum, Germany; 6 Eawag, Swiss Federal Institute of Aquatic Science and Technology, Department Surface Water Research and Management, Seestrasse 79, 6047 Kastanienbaum, Switzerland; 7 Institute of Biogeochemistry and Pollutant Dynamics, Swiss Federal Institute of Technology (ETH), 8092 Zürich, Switzerland; 8 Eawag, Swiss Federal Institute of Aquatic Science and Technology, Department Surface Water Research and Management, Überlandstrasse 133, 8600 Dübendorf, Switzerland; 9 Oeschger Centre for Climate Change Research, University of Bern, Falkenplatz 16, 3012 Bern, Switzerland; 10 Institute of Geochemistry and Petrology, Swiss Federal Institute of Technology (ETH), 8092 Zurich, Switzerland

## Abstract

In closed-basin lakes, sediment porewater salinity can potentially be used as a conservative tracer to reconstruct past fluctuations in lake level. However, until now, porewater salinity profiles did not allow quantitative estimates of past lake-level changes because, in contrast to the oceans, significant salinity changes (e.g., local concentration minima and maxima) had never been observed in lacustrine sediments. Here we show that the salinity measured in the sediment pore water of Lake Van (Turkey) allows straightforward reconstruction of two major transgressions and a major regression that occurred during the last 250 ka. We observed strong changes in the vertical salinity profiles of the pore water of the uppermost 100 m of the sediments in Lake Van. As the salinity balance of Lake Van is almost at steady-state, these salinity changes indicate major lake-level changes in the past. In line with previous studies on lake terraces and with seismic and sedimentological surveys, we identify two major transgressions of up to +105 m with respect to the current lake level at about 135 ka BP and 248 ka BP starting at the onset of the two previous interglacials (MIS5e and MIS7), and a major regression of about −200 m at about 30 ka BP during the last ice age.

## Introduction

**Figure 1:**
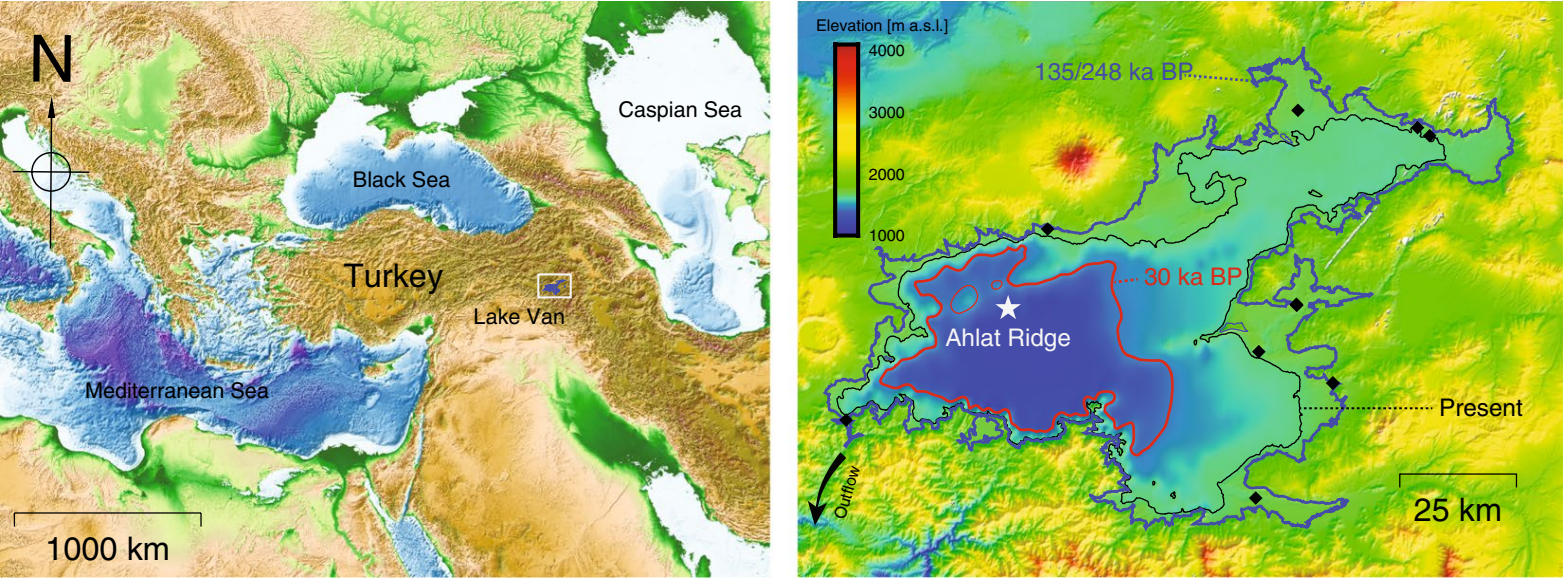
Left-hand panel: Overview map of eastern Europe and western Asia showing the location of Lake Van (open rectangle). The elevation data are from the Global Multi-Resolution Topography^51^. Right-hand panel: Map combining a high-resolution digital elevation model^42^ and the bathymetry of Lake Van^41^ used for the lake-level reconstruction in this work. The present lake level is plotted as a black contour line; past lake levels are plotted as colored contour lines (red: 30 ka BP; blue: 135 and 248 ka BP). The ICDP PaleoVan drill site at Ahlat Ridge is indicated by a white star. The spatial distribution of the terraces indicating past high lake levels^9–11^ (black rhomboids) agrees well with the area of maximum extension of Lake Van during MIS5e and MIS7. The maps of both panels of Fig. 1 were created using Generic Mapping Tools (GMT) version 5.3.0 (http://gmt.soest.hawaii.edu)^52^.

Lake Van (Turkey), with a depth of about 450 m and a volume of about 600 km^3^, is the largest soda lake on Earth and one of the largest closed-basin lakes (Fig. 1). Soda lakes are characterized by high concentrations of carbonate species (see the Results section for further details). This explains the lake’s present high pH of approximately 10 and high salinity of 23 g/kg^1–4^. The lake is known to respond very sensitively to changes in the hydrological regime of the surrounding region^5^, and is located at a key position for climate research between the Black Sea, Caspian Sea and Mediterranean Sea^6^. Hence, the sediments of Lake Van represent a valuable paleoclimate archive that span approximately 600 ka and cover several glacial/interglacial cycles^6, 7^.

Past lake-level changes with respect to the present lake level of 1645 m above sea level (a.s.l.) have been reported in studies of lake terraces on land^8–11^; in seismic^12, 13^, volcanological^14^, and sedimentological^15^ studies; and in studies of the geochemistry of the uppermost 10 m of the sediment column^3, 16, 17^.

Although the existence of lake terraces provides geomorphological evidence for high lake levels (up to +110 m above the current level, i.e. 1755 m a.s.l.; see the rhomboids in Fig. 1, right-hand panel), tectonic uplift or subsidence cannot be excluded in this region, which is located at the triple junction of the Eurasian, Afro-Arabian and Persian plates^1, 18, 19^. Further, the ages of these terraces are unknown or very poorly constrained, which limits their use for reconstructing the temporal evolution of the lake level.

Major regressions have been identified based on seismic reflection data^13^. Seismic transects across the basin of Lake Van reveal the presence of clinoforms and erosive channels, suggesting past low lake levels several hundred meters below the present lake level. However, the timing of the inferred regressions over the past 80 ka contrasts somewhat with the transgressions suggested by the age estimates of the terraces on land^9^, limiting any final assessment of past water volumes (or lake levels), and thus of the major hydrological states of Lake Van.

Earlier lake-level reconstructions by geochemical means in short sediment cores suggest that a maximum regression of more than −400 m from the present lake level occurred at about 15 ka BP (corresponding to a sediment depth of about 10 m, according to the age model adopted in the relevant publications), with a possible (but highly speculative) desiccation period^3, 20^. However, no indicators of desiccation, such as evaporites (e.g., aragonite crusts, dolomite) or iron oxides have been observed anywhere in the sedimentary sequence of Lake Van^6, 7, 15, 17^ and conformable strata, also in the deep basin, as seen in seismic data^6, 12^ show no evidence that the lake was ever completely dry.

Recent sedimentological investigations^7, 15^ provide additional temporal constraints for potential fluctuations in lake level, which are reflected in sharp lithological changes. Although these lithological changes cannot be converted directly into changes in lake level, some of them seem to be linked to the lake-level reconstructions based on seismic investigations and on the morphological analysis of terraces on land. The hypothesis of a rising lake level at the onset of the Holocene is supported by the existence of organic-rich laminae at about 12 ka BP. Well-stratified seismic facies indicate a relatively stable lake level close to its present state after 14 ka BP, following a pronounced low stand^13^. A major regression marked by lithologies typical of cold, dry climatic conditions around 15–27 ka BP correlates well with a regressive surface deposited 210 m below the present lake surface between 14 and 30 ka BP^13^. Geochemical and lithological evidence indicates rising lake levels from 84–83 ka BP (depositions similar to the recent sediments), 110–98 ka BP (sapropel-like layers), and 135–126 ka BP (laminae and presence of diatoms^15^). The ages of these deposits agree roughly with the ages of terraces on land located between 84 and 110 m above the present lake surface^9^.

**Figure 2:**
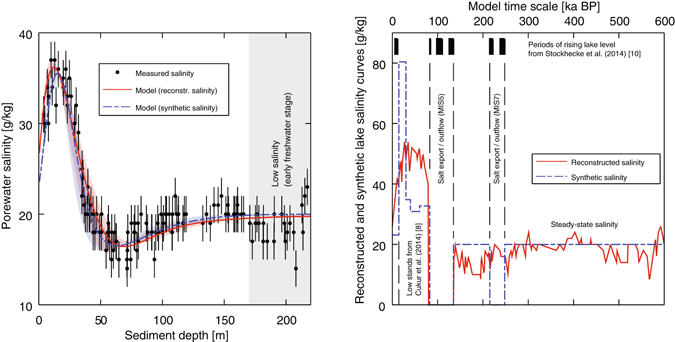
Salinity profile measured in the pore water of Lake Van. The porewater salinity was measured in core-catcher samples during ICDP PaleoVan drilling operations in 2010 (left-hand panel, black dots). The area shaded gray marks the depth range characterized by lower salinities that might correspond to past freshwater stages of Lake Van^15, 23^. Modeling the physical transport of salt through the sediment pore space (left-hand panel, red line and dashed blue line) shows that the salinity changes in the uppermost 100 m can be reproduced reasonably well either using the reconstructed (right-hand panel, red line) or synthetic (right-hand panel, dashed blue line) salinity curve as a temporal boundary condition at the sediment/water interface. The areas shaded red and blue in the left-hand panel represent the variation range of the model results depending on the molecular diffusivities of the various ions contributing to the overall salinity. Between 83 and 135 ka BP (MIS5), and between 215 and 248 ka BP (MIS7), the synthetic input curve implies the export of salt from Lake Van through an outflow.

To gain new insights into the hydrology of Lake Van, we present a reconstruction of the past major fluctuations in the lake volume and the corresponding lake level using the salinity (i.e., the total amount of dissolved solids per unit mass) measured in the pore water of core catcher samples collected at Ahlat Ridge (38.667°N, 42.670°E, water depth 357 m; black star in Fig. 1, right-hand panel) during drilling operations conducted as a part of the ICDP (International Continental Scientific Drilling Program, www.icdp-online.org) PaleoVan project^21–23^ (Fig. 2). The collected sediment cores cover the entire history of Lake Van since it first came into being as an endorheic water body at about 500 ka BP, as suggested by the higher CaCO_3_ concentrations determined in its sediments since MIS14^15^.

Our lake-level reconstruction using porewater salinity is based on simple and straightforward physical concepts. During sedimentation, part of the water overlying the sediments is buried in the pore space^24, 25^. If a concentration signal persists in the water body for sufficiently long, the signal can be archived in the sediments^25^. Insofar as the amount of dissolved solids in a terminal lake is close to steady-state^26, 27^, the salinity of the lake water is inversely proportional to the volume of water present in the lake basin. Thus, as the morphometry of the lake basin is known, changes in porewater salinity can be converted directly into changes in lake level. As the water balance of Lake Van is controlled mainly by river discharge and evaporation, the lake-level reconstruction presented here also provides insights into past precipitation regimes in the lake catchment^5, 28^. It should be noted that our reconstruction accounts only for long-term changes in lake level (i.e., over tens of thousands of years^25^) and is not suitable for reproducing events on shorter time scales, as short-term fluctuations are expected to be smoothed out by diffusion in the pore space^25^.

Reconstruction of lake volumes and the respective lake level changes from porewater salinity implicitly assumes that salinity behaves conservatively, i.e. that it is not affected by secondary alteration processes. In the following paragraph we briefly list key arguments that imply the porewater salinity of Lake Van to be geochemically conservative.

According to the insights provided by noble-gas geochemistry in the pore water^23^, the presence of groundwater or secondary processes significantly affecting the conservative behavior of porewater salinity at Ahlat Ridge can be excluded. For instance, the typical excess air signature^29, 30^ produced by the forced dissolution of air bubbles trapped in the pore space during water table fluctuations commonly found in aquifers is absent^23^. Furthermore, measured and modeled He concentration profiles in the pore water at Ahlat Ridge agree with each other^23^. This agreement indicates that the observed non-atmospheric He concentrations are only the result of local production and accumulation within the sediment column. Thus, we conclude that the pore water originates only from the overlying water body, and that the pore space is not affected (significantly) by groundwater. Recently, we proposed noble-gas solubility equations^4^ tailored to the specific water chemistry of Lake Van that allow past environmental conditions to be reconstructed^29, 31^ (e.g., salinities calculated from measured noble-gas concentrations, also known as “noble-gas salinities”). The estimated noble-gas salinities agree rather well with the salinities measured in the pore water^23^. Such agreement offers strong support for the argument that the measured porewater salinity constrains noble-gas solubility during air/water partitioning and deep-water mixing in Lake Van. This indicates that secondary alterations of porewater salinity (e.g., by diagenetic processes) are of minor importance, and that porewater salinity is hence virtually geochemically conservative, as there would otherwise be a discrepancy between noble-gas salinities and pore water salinities.

## Results

### Salinity steady-state in Lake Van

From the available hydrochemistry data^3, 5^ it is possible to estimate the steady-state concentrations of solutes in the bulk water of Lake Van (see Methods). It should be noted that the special soda chemistry and high pH of the lake means that the ion balance in its water is dominated by Na^+^, K^+^, ${{\rm{HCO}}}_{3}^{-}$, ${{\rm{CO}}}_{3}^{2-}$, and Cl^−^. At present, the proportion by mass of Cl^−^ with respect to the total mass of dissolved salts is similar to that of Na^+^ (about 25%) but lower than that of the carbonate species (about 40%). The estimated accumulation times^3^ of Na^+^ (99 ka), K^+^ (28 ka) and all carbonate species (7 ka) indicate that these dissolved components reach their steady-state concentrations much faster than Cl^−^ (201 ka). Hence, Cl^−^ represents the “worst case scenario” when assessing the presence of a steady-state in the total salinity.

The expected steady-state Cl^−^ concentration in Lake Van is 7.6–10.2 g/L, which should be attained within several 100 ka. This implies a certain “inertia” of the whole lake system, which limits the temporal resolution of our salinity and lake-level reconstructions. The Cl^−^ concentration in the rivers is expected to vary both seasonally and annually. However, no comprehensive data set on the major-ion composition of the water of the rivers feeding Lake Van exists. Hence, the riverine Cl^−^ input is probably biased by seasonal effects^3^ and variability on longer time scales. Our simple model for the Cl^−^ budget should therefore be considered as a “best guess”.

The measured Cl^−^ concentration in Lake Van of about 6.3 g/L^5^ suggests that 60–80% of the steady-state concentration has been reached. The assumption that the Cl^−^ concentration is close to steady-state is supported by the almost constant porewater salinity of approximately 20 g/kg found below a sediment depth of 100 m, and by the decrease in salinity in the uppermost 16 m of the sediment toward similar values at the sediment/water interface (Fig. 2). The absence of steady-state should have resulted in a general salinity increase over time - which is not observed in the porewater salinity profile below 100 m. The decrease in salinity in the upper few meters of the sediment column is supported by previously determined chloride concentration profiles showing a similar trend^3^. This suggests that salinity fluctuations represent only transient states of Lake Van around a geochemical steady-state. Hence, past and present salinities of about 20 g/kg most likely result from the long-term balance between the salt input from rivers and the salt export resulting from burial in the sediments at an average sedimentation rate of 0.37–0.50 mm/a (see Methods^3, 15, 16, 22^). Our steady-state calculation indicates that processes other than sedimentation are of minor importance for the salt export from Lake Van.

**Figure 3:**
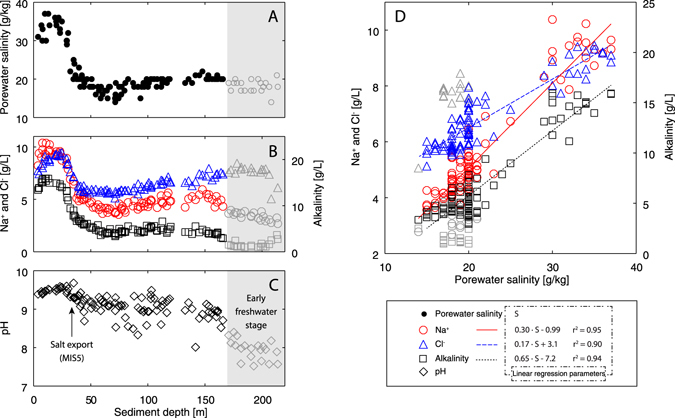
Salinity, Na^+^, Cl^−^, alkalinity, and pH profiles measured in the pore water of Lake Van. All measurements were conducted in the pore water of the same core-catcher samples used for the determination of salinity. The curves of the conservative species Na^+^ (panel B, red circles) and Cl^−^ (panel B, blue triangles), as well as that of alkalinity (panel B, black squares), mimic the shape of the salinity profile (panel A; see also Fig. 2). The linear correlations that exist between salinity and both Na^+^ and Cl^−^ (panel D, red circles/line and blue triangles/line) demonstrate that total salinity in Lake Van is virtually geochemically conservative and can thus be used as a conservative tracer for reconstructing lake levels. The linear correlation between salinity and alkalinity (panel D, black squares/line) indicates the presence of stable hydrochemical conditions over the time span relevant for the inferred lake-level changes. The gray symbols indicate measurements conducted in porewater samples with lower salinities that might represent a relict of Lake Van's earlier, freshwater, stage^15, 22^; these measurements were therefore excluded from the calculation of the least squares regressions in panel D. The mean pH value for the time span relevant for the lake-level reconstruction is 9.1 ± 0.3 (calculated using the data plotted as black circles in panel C). This rather constant pH again suggests that the composition of the dissolved species in the water body of Lake Van was rather stable. A slight drop in pH observed at 32 m (approximately 86 ka BP) coincides with a water outflow (“open condition”) exporting salt from the lake during MIS5. A major decrease in pH below 170 m (gray circles) seems to result from the entrapment of “paleowater” from the time when the basin of Lake Van was not yet closed and operated as a freshwater system.

The salinity profile agrees reasonably well with the measured Na^+^, Cl^−^, and alkalinity concentration profiles (Fig. 3, panels A and B). Both conservative species Na^+^ and Cl^−^, as well as alkalinity, correlate linearly with salinity (Fig. 3, panel D). The measured pH profile (Fig. 3, panel C), showing relatively stable values around an average of 9.1 ± 0.3 down to 170 m, suggests the presence of rather stable hydrochemical conditions over several hundred thousand years. This observation supports the assumption that, at least for the time span relevant to our reconstructions, salinity in Lake Van is rather conservative and close to compositional steady-state, and that the observed salinity minima and maxima can be interpreted as real geochemical features produced by changes in lake volume.

### Solute transport in the pore space

Similarly to the approach adopted to reconstruct past salinities in the ocean^32–36^ and in the Dead Sea^37^, we applied an advection-diffusion model^25^ to obtain a better understanding of the physical transport processes responsible for the observed salinity profile in the sediment column of Lake Van. The physical transport model for solutes in the pore space allows a rough estimate to be obtained of the original salinity in the water body at the time of porewater formation by correcting the measured salinity (*S*_m_) in the pore water in the uppermost 100 m of the sediment column by using peak amplification by a given factor (*x*) around the assumed steady-state value of 20 g/kg (*S*_st_) to compensate for the smoothing produced by diffusive exchange (i.e., *S*_rec_ = *S*_m_ + *x* · (*S*_m_ − *S*_st_), whereby *S*_rec_ is the reconstructed salinity).

In our model the reconstructed salinity curve (Fig. 2, right-hand panel, red line) was determined as an upper boundary condition at the sediment-water interface over a time span of 600 ka (i.e., the salinities were linearly interpolated and distributed over the chosen time span). The model implicitly assumes that the reconstructed salinity curve is representative of the whole water body; i.e., that the water column was well mixed on the relevant time-scale of several millennia. Such homogeneous conditions are supported by recent limnological investigations^5, 19^ and by sedimentological evidence^15^ indicating that Lake Van had frequent phases of complete mixing. It should be noted that the ages in the modeling exercise refer to the time of deposition of the solid (matrix) sediment phase. A sedimentation rate of 0.37 mm/a (i.e., 220 m/600 ka^15^), a terminal porosity of 60%^38^ and a molecular diffusivity of 1.5 · 10^−9^ m^2^/s (a value typical for strong electrolytes representing the average of the diffusivities of the major ions^39^ contributing to salinity) were chosen. The assumption of a constant sedimentation rate is supported by the almost linear increase in sediment age with increasing sediment depth^7^. Nevertheless, deviations from the assumed sedimentation rate should be considered as a potential source of uncertainty in modeling the solute transport. Previous modeling of the production and transport of terrigenic He^23^ in Lake Van relied also on a constant porosity profile. As the terrigenic He production rates depend strongly on the chosen porosity (because they are partially determined by the ratio of the volume of sediment matrix hosting the parent radionuclides U and Th to the porewater volume), the agreement between the modeled and the measured He concentrations indicates that the adopted constant porosity is an acceptable approximation to the real porosity profile of the investigated sediment column. In line with a recent determination of the solute transport properties of the shallow sediments of Lake Van, the effective diffusion coefficient in the pore space was set to 10% of the molecular diffusion coefficient^38^. Our recent analysis of the transport of terrigenic He in the deep sediments of Lake Van demonstrates that such attenuation of diffusivity is also valid for more compacted sediments, and thus also for the present modeling exercise^23^. Indeed the numerical model applied in the mentioned work^23^ allowed the measured He concentration profiles to be reproduced over a time span of 600 ka, demonstrating that the adopted constant effective diffusivity is a reasonable approximation for both the shallow and the deep sediments of Lake Van. Discrepancies between assumed and real effective diffusion coefficients might affect the modeling results. On the one hand, the effective diffusion coefficient directly influences the conservation of concentration signals in the pore space (i.e., minima/maxima are better preserved with lower *D*_eff_). On the other hand, in the presence of high concentration gradients produced by salinity minima and maxima occurring very close to each other, *D*_eff_ determines the extent to which the respective concentration signals mix (which can, for example, result in an apparent drift of the two concentration peaks in opposite directions). Deviations of the molecular diffusivity of the individual species from the average molecular diffusivity do not significantly affect model predictions (see Fig. 2, left-hand panel, red and blue shaded areas). The maximum deviation, that for ${{\rm{CO}}}_{3}^{2-}$, is 6 · 10^−10^ m^2^/s, which results in a difference in effective diffusivity of 6 · 10^−11^ m^2^/s. The low effective diffusion coefficient of the sediments of Lake Van seems to limit fractionation of the dissolved species and to foster a geochemically conservative behavior of salinity. As the reconstructed salinity curve results from the simple scaling of the measured salinities, the age uncertainty is mainly given by the diffusive mixing of the original concentration signals over time. This error can be scaled using the 2*σ* (95%) interval of the diffusive mixing length $L=\sqrt{{D}_{{\rm{eff}}}t}$ (where *D*_eff_ is the effective diffusivity [m^2^/s] and *t* is time [s]). This characteristic length can be converted into ages by using the constant sedimentation rate adopted for the modeling (see below). The respective uncertainties range from 9 ka (topmost salinity at 4 m) up to 67 ka (bottom salinity at 214 m). Applying the reconstructed salinity input curve allows the measured salinity to be reproduced fairly well in the uppermost 100 m (Fig. 2, left-hand panel, red line).

The measured salinities within the depth range of 170–220 m (Fig. 2, left-hand panel, grey shaded area) are significantly lower than the present salinity of the lake water. Such lower salinities suggest that even the deep sediments of Lake Van might have trapped porewater salinity signals on time-scales exceeding 250 ka by further attenuation of the effective diffusivity in the pore space to an extent where diffusive processes became negligible^23, 24, 40^. This interpretation is supported by the Na^+^, Cl^−^, alkalinity, and pH measurements in the deepest part of the sediment column (Fig. 3), which also provide strong evidence for the entrapment of less saline “paleowater”. We speculate that the observed lower salinities represent a relict of Lake Van’s earlier, freshwater, stage^15, 22^. Hence, the observed concentration peaks below 100 m might indeed be real features resulting from lake-level fluctuations that, however, do not allow a quantitative estimate of past lake levels.

The reconstructed salinity curve indicates that the highest salinity originally deposited in the pore water of the sediments at a depth of about 16 m (about 40 ka BP) was approximately 50 g/kg. To reproduce the low salinities between 50 and 80 m with a distinct salinity minimum at about 65 m, the advection-diffusion model would ultimately require the lake water to be virtually salt-free during 80–135 ka BP. The drop in the salinity of the lake water required to reproduce the measured low porewater salinity indicates that during a significant length of time (or during many consecutive periods of time with minor interruptions), Lake Van had an outflow that exported salt from the lake basin rapidly enough to allow freshwater conditions to become established in the water body.

The results of our salinity modeling can be further improved and refined by constructing a synthetic salinity curve based on external evidence (Fig. 2, right-hand panel, dashed blue line) that follows the reconstructed salinity curve and is proportional to the changes in water volume in relation to the major and most persistent regressions and transgressions over the last 250 ka. The upper limit of the synthetic salinity curve between 0 and 83 ka BP is calculated from the low stands identified by seismic profiling^13^. The respective lake levels^13^ represent only the past water level relative to the present lake level; they have been converted into water depths compatible with the present morphology of Lake Van by adding the thickness of sediments accumulated since the deposition of the observed seismic features. Average water volumes for each relevant period of time have been calculated from the water depths using the bathymetry of Lake Van^41^. The values of the synthetic salinity curve back to 83 ka BP are inversely proportional to the changes in the volume of the lake under the assumption that the total mass of dissolved salts remained constant. Although this assumption is supported by our steady-state calculations, the latter are based on a very limited amount of available data. Past major changes in the lake volume could have modified drainage patterns and affected to a certain extent the total mass of dissolved salts. These factors should be considered as unquantifiable uncertainties affecting our steady-state estimations. Periods of freshwater conditions in the lake are synchronous with the timing of rising lake levels suggested by sedimentological information^15^. The synthetic salinity curve indicates that the highest salinity originally deposited in the pore water of the sediments approximately 30 ka ago was about 80 g/kg. The uncertainty in the ages of the synthetic salinity curve is directly related to the precision of the dating performed in a recently published work^7^. The respective age errors (≤2.6 ka) have only a minor effect on the salinity modeling. The results of the modeling using the synthetic salinity curve (Fig. 2, left-hand panel, dashed blue line) allow an additional earlier freshwater stage to be identified: to reproduce the low salinities between 50 and 80 m a salt-free water mass must have been present in the lake during 83–135 ka BP and 215–248 ka BP.

We note that the integrals of the reconstructed salinity curve and the synthetic salinity curve from 0–100 ka BP are very similar (see Fig. 2, right-hand panel). As both curves are subject to the same signal damping by vertical diffusion in the sediment, both salinity boundary conditions reproduce the measured salinity profile equally well. This illustrates that the salinity profile observed in the sediment of Lake Van is sufficient to provide a continuous reconstruction of past lake levels. The additional information on short-term lake-level evolution provided by discontinuous, external features such as terraces and clinoforms is consistent with the continuous reconstruction of the lake level from the porewater salinity profile.

### Lake-level reconstruction

**Figure 4:**
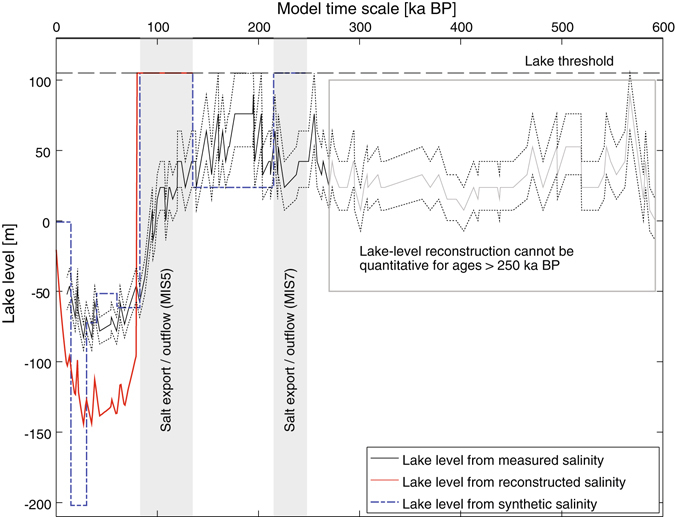
Lake-level reconstruction for Lake Van. Changes in lake level with respect to the present lake level at 1645 m a.s.l. (solid black line) were calculated from salinity measurements in the pore water of ICDP PaleoVan core-catcher samples (Fig. 2, black dots). Note that the model time scale (x-axis) refers to changes in salinity in the open water column, which sets the top hydrochemical boundary condition for the modeling exercise (Fig. 2). Dotted lines represent the error range given by the precision of the salinity measurements. The red line shows the lake level calculated from the reconstructed salinity curve (Fig. 2, right-hand panel, red line). The dashed blue line depicts the lake level based on the synthetic salinity curve (Fig. 2, right-hand panel, dashed blue line) and on seismic and sedimentological information. The salinity minimum between 50 and 80 m (Fig. 2, left-hand panel) can be interpreted as two high lake stands +105 m above the current lake level at an altitude of 1750 m a.s.l., where the basin morphology allowed Lake Van to overflow. For ages beyond 250 ka the lake-level reconstruction cannot be interpreted quantitatively (gray line), as the measured salinity most likely reflects the long-term steady-state salinity of the lake.

We combined a high-resolution digital elevation model^42^ and the bathymetry data for Lake Van^41^ to obtain a three-dimensional representation of the lake catchment, which we then used to calculate the volume of water (and the corresponding lake level) necessary to produce the variation in dissolved salt concentration that would be necessary to yield both the measured and reconstructed salinity profiles. The measured salinity profile, which results from the smoothing of the initial salinity concentration signals in the water body by diffusion in the sediment pore water, can be used to infer the minimum lake-level changes (Fig. 4, black line). The reconstructed salinity curve, which represents the original salinity in the water body, can be used to infer the average long-term amplitudes of the reconstructed lake-level changes (Fig. 4, bold red line). The synthetic salinity curve reflects the most likely timing of the lake-level changes based on the latest seismic and sedimentological information (Fig. 4, bold dashed blue line).

The lowest stand of Lake Van inferred from our reconstruction using the reconstructed and synthetic salinity curves lay between −145 and −200 m with respect to the present lake level (red contour lines in Fig. 1, right-hand panel). This low stand occurred at about 14–30 ka BP, coinciding with the occurrence of dry conditions in eastern Anatolia during the last glacial period^15^. It should be noted that the results of the advective-diffusive model using either of the salinity inputs can only reproduce the measured salinity maximum if a general low stand between −50 and −70 m persisted between 30 and 83 ka BP, as suggested by the shape of the reconstructed salinity curve and the seismic interpretations^13^. Therefore, the possibly coeval terraces on land^9^ seem to represent only short-term high stands (i.e., changes that do not significantly affect porewater salinity on the time scales relevant to our modeling). Our results suggest that between 14 and 83 ka BP the volume of the lake was rather small (less than 50% of the present volume) and the lake level was fairly low.

The analysis of the reconstructed salinity curve implies that the highest stand of Lake Van was at least +105 m above its present level; i.e., it was about 1750 m a.s.l. (see the blue contour lines in Fig. 1, right-hand panel). In line with the interpretation of the salinity minimum at about 65 m, according to the digital representation of the catchment^41, 42^ such a high lake level indicates that for some time Lake Van had an outflow located in the south-western part of the basin^9^ that allowed salt to be exported from the water body into the upper Tigris drainage basin (the location of the outflow is indicated by a black arrow in Fig. 1, right-hand panel). This hypothesis of an “open” Lake Van 80–135 ka ago is supported by the presence of undated lake terraces located at 1751 m a.s.l. and by the occurrence of unbroken shells of *Dreissena* sp. (the zebra mussel, an organism often found in freshwater systems) at an altitude of 1740 m a.s.l.^9^. The highest dated terraces, at 1726 m a.s.l., have a minimum age of about 112–119 ka^9^, which corresponds to Marine Isotope Stage 5 (MIS5). In the light of our modeling exercise to reconstruct the evolution of the salinity of the lake, we are tempted to relate the highest undated terraces to the major transgression identified using our reconstructed and synthetic salinity curves, which were constructed based on sedimentological evidence for freshwater-like conditions during 126–135 ka BP and high stands during 83–84 ka BP and 98–110 ka BP^15^. Hence, these terraces might have an age of 110 ± 30 ka if we consider the salinity export needed in our model to reproduce the salinity minimum measured at 65 m. Despite the fact that our modeling is not suitable for inferring accurate ages, sedimentological evidence supports the concept that a salt export from Lake Van most likely occurred at the onset of MIS5, which is known to have been a rather humid period in eastern Anatolia^43^.

Analysis of the synthetic salinity curve also allows the identification of an “open state” of Lake Van during MIS7, where sapropel-like layers observed in the ICDP PaleoVan sediment cores suggest periods of high lake level (215–248 ka BP^15^). The existence of an earlier freshwater stage improves the reproduction of the intensity and depth of the observed salinity minimum at about 65 m, which cannot be achieved adequately if MIS5 is considered as the only period with freshwater conditions in the synthetic salinity curve. As a consequence of the strong diffusive smoothing over 250 ka of the high concentration gradients produced by the inferred freshwater stages, the volume of Lake Van between MIS5 and MIS7 cannot be assessed in our modeling exercise. However, based on the immediate increase in salinity observed after MIS5, we speculate that during MIS6 salinity was again close to steady-state. The fact that salinity must have recovered much more quickly than predicted by our steady-state calculations underlines the current very limited knowledge of the past discharge and chemical composition of the rivers feeding Lake Van.

## Discussion

In this study, for the first time, lake levels have been reconstructed in a quantitative manner based on sediment porewater salinity data. Porewater salinity is shown to be a simple and relatively straightforward geochemical proxy that allows the past hydrology of Lake Van to be reconstructed over a time span of about 250 ka. In particular, a major low stand corresponding to a lake volume less than half the present lake volume occurred during the last glacial, whereas during MIS5 and MIS7 the volume of the lake was five times greater than it is today. Such volume estimations are possible because the total mass of dissolved salts in the water body of Lake Van is close to steady-state, as shown by our simple mass-balance model (which seems to be applicable to other closed-basin systems and to the ocean; see Methods).

It is not possible to reconstruct directly the exact timing of past salinity changes from the porewater profile. Nevertheless, lake-level histories proposed by other studies are in line with our results, providing a consistency check for the method applied to reconstruct past lake volumes from porewater salinity.

Our results are supported by the outcome of a recent global hydroclimate reconstruction that combines various high-resolution proxy records form Lake Van and simulations based on the LOVECLIM earth system model^44^. In this reconstruction, millennial-scale droughts, such as the one identified in our work at about 30 ka BP, were found to coincide with Dansgaard-Oeschger stadials during MIS2 to MIS4. Also, an increase in precipitation in the Eastern Mediterranean that is consistent with the timing of the inferred high stands at about 135 and 248 ka BP, has been shown to correspond to higher sea surface temperatures in the North Atlantic^44^. The outcome of our study thus suggests that, on long time-scales, the salinity of Lake Van is likely to be directly linked to large-scale climate forcing.

Compared to previous seismic, volcanological, sedimentological, geochemical, and geomorphological studies, the modeling of porewater salinity is the only scientific approach that leads to the unambiguous identification of an outflow from Lake Van during major high stands. Our findings underline the potential of porewater salinity as an additional, direct proxy for the reconstruction of past lake volumes and the related lake levels of other closed-basin, saline water bodies (e.g., the Caspian Sea and Lake Issyk-Kul^24^).

## Methods

### Salinity and pH measurements

During the ICDP PaleoVan drilling operations^6, 21^ in Lake Van in 2010, the salinity of the lake water and of the pore water in core-catcher samples was measured with a refractometer (VWR, Item No. 635–0171) with an accuracy of ±2 g/kg. The refraction index of a water sample is proportional to its density, and thus is directly related to its true, absolute salinity^45^ (i.e., the salinity based on the total mass of all dissolved solids, whether ionic or nonionic). Pore water for the salinity measurements was extracted from the bulk sediment with a GEOTEK porewater squeezer (http://www.geotek.co.uk) and filtered through a glass-fiber prefilter (Sartorius, Item No. 13400–100, diameter 100 mm) and a hydrophilic polyethersulfone membrane (VWR, Item No. 514–4233, pore size 0.45 μm, diameter 25 mm). pH was determined in the field simultaneously to the salinity measurements using a portable pH meter (Metrohm Model 704).

### Na^+^, Cl^−^, and alkalinity measurements

Na^+^ concentrations were determined in porewater aliquots acidified with 2% suprapure ${{\rm{HNO}}}_{3}^{-}$. The analyses were carried out at the Swiss Federal Institute of Aquatic Science and Technology (Eawag) using an Arcos ICP-OES (internal standard deviation < 1%) after 1000-fold dilution. Cl^−^ concentrations were measured with an ICS-2100 ion chromatograph (DIONEX) after 100-fold dilution. Alkalinity was determined by titration at the Analysis and Education Lab at Eawag.

### Salinity steady-state calculation

The salinity steady-state calculation is based on published data on the geochemical composition of the dissolved species in the water of Lake Van^3, 5^ and on our Cl^−^ concentration measurements in the major inflows, the Bendimahi River (3.4 mg/L) and the Zilan River (4.7 mg/L). We used the following mass balance of dissolved salts in the water body to calculate salinity changes:

1 $$\frac{\partial ({V}_{tot}\cdot S)}{\partial t}={Q}_{in}\cdot {S}_{in}-{Q}_{sed}\cdot S$$

where *V*_*tot*_ is the volume of Lake Van [m^3^], *S* is the salinity in the lake water [g/kg] (alternatively [g/L]), *t* is time [s], *Q*_*in*_ is the riverine water discharge [m^3^/s], *S*_*in*_ is the river salinity [g/kg] (alternatively [g/L]) and *Q*_*sed*_ = *r*_*sed*_ · *A* · *ϕ* is the porewater flux into the sediments [m^3^/s], which takes into account the sedimentation rate *r*_*sed*_ [m/s], the area of Lake Van *A* [m^2^], and the terminal sediment porosity *ϕ* [%]^25, 46^. The salinity steady-state is reached when ∂(*V*_*tot*_ · *S*)/∂*t* = 0 and hence

2 $$S=\frac{{Q}_{in}}{{Q}_{sed}}\cdot {S}_{in}$$

In the modeling exercise we assumed the following variables to be constants characteristic of present conditions in Lake Van: *Q*_*in*_ = 2 km^3^/a^3^; *r*_*sed*_ = 0.37–0.50 mm/a^3, 16, 22^ (the lowest sedimentation rate here is derived from the value 220 m/600 ka that was obtained during a recent investigation of the ICDP drill cores^15^); *A* = 3600 km^2^; and *ϕ* = 60%^38^. With an average Cl^−^ concentration of 4.1 mg/L in the inflows, the expected steady-state Cl^−^ concentration in Lake Van is about 7.6–10.2 g/L. The characteristic time scale involved in attaining steady-state is determined by the mean residence time of dissolved salts in the water body, which is given by:

3 $${t}_{s}=\frac{S\cdot {V}_{tot}}{{S}_{in}\cdot {Q}_{in}}$$

For Cl^−^ in Lake Van *t*_*s*_ is estimated to be 440 ka. The characteristic times required for Na, K, and alkalinity (i.e., carbonate species) to reach steady-state can be similarly inferred from the mean measured concentrations of each in the lake water (6.6 g/L, 0.5 g/L, and 10 g/L, respectively) and the river water (14.6 mg/L, 3.8 mg/L, and 166 mg/L, respectively). It turns out that *t*_*s*_ is 136 ka for Na^+^, 40 ka for K^+^, and 18 ka for the carbonate species. As the lake became an endorheic water body at about 500 ka BP, the total salinity nowadays is assumed to be close to steady-state. It should be noted that this age coincides with a phase of substantial change in the geomorphology of the lake basin, which had previously hosted an open freshwater body. Since approximately 500 ka BP, Lake Van has therefore been characterized by a completely different hydrogeochemistry; viz. that of a salt lake^4, 5^.

The model presented above also seems to work, at least in terms of a zeroth-order approximation, in the case of the ocean - the largest closed-basin system on Earth. If the recent ocean salinity is assumed to be at steady-state, as is commonly accepted, then the input and output parameters to calculate the salinity steady-state can be used to compare the input of salt to, and the loss of salt from, the ocean (e.g., to relate the input of salt from rivers to its burial by sedimentation). Based on the model presented above, it is possible to estimate roughly the average global sedimentation rate in the ocean as 0.5–0.8 mm/a, assuming *Q*_*in*_ = 3.7 · 10^4^ km^3^/a^47^; *S*_*in*_ = *M*_*in*_/*Q*_*in*_ = 0.11 g/kg, where the annual mass of discharged salts *M*_*in*_ = 4 · 10^12^ kg/a^48^; *A* = 3.6 · 10^8^ km^2^; and *ϕ* = 40–60%. Such a sedimentation rate lies reasonably well within the range of sedimentation rates known for coastal and pelagic regions. With *V*_*tot*_ = 1.3 · 10^9^ km^3^ ^49^ the respective *t*_*s*_ (see Eq. 3) is about 12 Ma. The calculated *t*_*s*_ is significantly shorter than the age of the oceanic crust^50^. The depositional environment required for porewater burial therefore seems to be guaranteed. This suggests that the salinity of the ocean might be the net result of the global salt input by rivers and the salt loss by sedimentation. In combination with the diffusive smoothing of concentration signals, this long response time of Cl^−^ in the ocean yields a reasonable explanation of why particular conditions (e.g., lithologies with low permeabilities, locally high sedimentation rates)^40^ are necessary to reconstruct past ocean salinities on glacial/interglacial time-scales using salinity profiles measured in the pore water of ocean sediments.
